# VH3810109 (N6LS) broadly neutralizing antibody safety, pharmacokinetics, and anti-drug antibody incidence in adults without HIV: phase 1 SPAN study results

**DOI:** 10.1128/aac.00258-25

**Published:** 2025-07-23

**Authors:** Peter A. Leone, Jan Losos, Paul Wannamaker, Riccardo D’Agostino, Michael Warwick-Sanders, Gabriela L. Ghita, Viviana Wilches, Christina Donatti, Kathryn Brown, Yash Gandhi

**Affiliations:** 1 ViiV Healthcare, Durham, North Carolina, USA; 2 GSK, London, United Kingdom; 3 GSK 525885 , Collegeville, Pennsylvania, USA; 4 ViiV Healthcare 252349, London, United Kingdom; 5 Certara 205185 , Radnor, Pennsylvania, USA; IrsiCaixa Institut de Recerca de la Sida, Barcelona, Spain

**Keywords:** ADA, bNAb, injection site reaction, N6LS, NRS, pharmacokinetics, safety

## Abstract

The CD4-binding site antibody VH3810109 (N6LS) demonstrated broad and potent neutralization activity *in vitro* and robust antiviral activity in people with HIV-1. We report safety, tolerability, pharmacokinetics, and anti-drug antibody (ADA) incidence for subcutaneous (SC) and intravenous (IV) N6LS. Safety and pharmacokinetics of a single dose of VH3810109 (also known as GSK3810109), administered either subcutaneously (SC) with rHuPH20 or intravenously (IV) [SPAN]) was an open-label, three-part, phase 1 study evaluating single-dose N6LS in adults without HIV (part 1, 20 mg/kg SC + recombinant human hyaluronidase PH20 [rHuPH20] 2,000 U/mL; part 2, 60 mg/kg IV; part 3, 3,000 mg SC + rHuPH20 2,000 U/mL). Over 24 weeks, adverse events (AEs), injection site reactions (ISRs), pharmacokinetics, and ADAs were monitored. Twenty-four participants (8/part) were enrolled and received a single N6LS dose. Overall AE incidence between SC doses was similar and higher compared with IV, driven by ISRs. No ISRs were reported for IV N6LS; for SC, 15/16 participants reported ISRs, and 17/32 events were grade 3 (all injection site erythema). All ISRs resolved without sequelae or treatment. All participants rated local reactions and pain as acceptable. No serious AEs or deaths occurred. Pharmacokinetics were as expected for a broadly neutralizing antibody; median terminal half-life ranged from 43 to 47 days. No ADAs were observed after IV N6LS (0/8); 5/16 participants had treatment-emergent ADAs after SC N6LS; however, a clear impact on pharmacokinetics was observed in only one participant. N6LS administered IV or SC + rHuPH20 had a favorable safety profile and was well tolerated. Results support the ongoing development of N6LS 3,000 mg SC + rHuPH20 and 60 mg/kg IV into phase 2b.

**CLINICAL TRIALS:**

Registered at ClinicalTrials.gov (NCT05291520).

## INTRODUCTION

Advances in antiretroviral therapy (ART) have led to worldwide declines in HIV incidence and AIDS-related mortality over recent decades, but HIV treatment remains a lifelong commitment (https://www.unaids.org/en/resources/fact-sheet). The continued development of ART with diverse mechanisms of action can provide more treatment options for people with HIV-1 whose needs are not met with current regimens, including those who prefer less frequent dosing (vs daily oral ART) or who have a limited number of fully active drug classes available.

Broadly neutralizing antibodies (bNAbs) bind to specific regions of the HIV-1 envelope glycoprotein trimer to block target cells from infection, several of which have been investigated as long-acting HIV-1 therapy or prevention in clinical studies or are currently being developed (1–7). The bNAb VH3810109 (N6LS; also known as GSK3810109) binds to the CD4-binding site of the envelope protein and has demonstrated broad neutralization activity *in vitro* and potent antiviral activity in non-human primate models of HIV-1 (8, 9). N6LS and other bNAbs have shown exposure-dependent antiviral activity, such that greater antiviral responses can be obtained with higher exposures (3, 10). In the phase 2a Broadly neutralizing antibody N6LS in ART naives to evaluate virologic response (BANNER) study (ClinicalTrials.gov, NCT04871113), N6LS led to virologic response in 93% of participants with no prior exposure to ART receiving a single dose at either 40 mg/kg or 280 mg (~4 mg/kg) administered intravenously (IV) and demonstrated a good safety profile across all doses (70 mg [~1 mg/kg] to 40 mg/kg IV and 700 mg [~10 mg/kg] subcutaneously [SC]) (11, 12).

The phase 1 SPAN study (ClinicalTrials.gov, NCT05291520) aimed to evaluate the safety, tolerability, and pharmacokinetics (PK) of single-dose N6LS in adults without HIV to support clinical studies of multiple-dose N6LS in adults with HIV-1. Relatively high flat doses and weight-based doses of N6LS were selected for the SPAN study to ensure that exposures achieved would provide sufficient safety, tolerability, and PK data from adults without HIV to support exposures expected to be explored in subsequent studies in adults with HIV-1. Volumes greater than typically used for SC injections (>1.5 mL) with standard delivery methods (13) were required to deliver the SC doses assessed in this study. Therefore, SC doses of N6LS were co-administered with recombinant human hyaluronidase PH20 (rHuPH20), an enzyme approved by the U.S. Food and Drug Administration as the active pharmaceutical ingredient in Hylenex (Halozyme Therapeutics, Inc., San Diego, CA) (14) and as part of several combination products (15) to increase the dispersion and absorption of other SC-injected drugs by depolymerizing the hyaluronan gel barrier against bulk fluid flow in the SC space. rHuPH20 facilitates the administration of the N6LS volumes needed to achieve each SC dose (16). Here, we report safety, tolerability, PK, and anti-drug antibody (ADA) data in adults without HIV from the SPAN study, which evaluated single N6LS doses of 20 mg/kg SC (~15 mL, with rHuPH20), 3,000 mg SC (~40 mg/kg; ~30 mL, with rHuPH20), and 60 mg/kg IV.

## MATERIALS AND METHODS

### Study design and population

SPAN was an open-label, three-part, 24 week, phase 1 study evaluating N6LS administered SC + rHuPH20 or IV in adults without HIV, with the intention of collecting data to inform dosing in future studies in adults with HIV-1. SPAN was conducted at a single site in the United States (PPD, Austin, TX). Participants were recruited from PPD listings and enrolled between February 2022 and April 2023.

Participants received either a single 20 mg/kg (~15 mL) SC dose of N6LS + rHuPH20 (part 1), a single 60 mg/kg IV dose of N6LS (part 2), or a single 3,000 mg (~40 mg/kg; ~30 mL) SC dose of N6LS + rHuPH20 (part 3). The enzyme rHuPH20 was co-administered with SC doses at 2,000 U/mL (maximum of 60,000 U); SC doses were administered in the abdomen. The infusion rate for SC dosing was 3 mL/min, and IV infusions were administered over ~60 min. Flow rates used for IV infusions varied since the dose (and thus the volume) was based on participant weight and added to a 250 mL IV bag. Part 1 dosing was used previously (16). To mitigate the risk of unexpected safety events for the higher untested doses in parts 2 and 3, study treatment was initially administered to a cohort of two participants each; in part 2, the remaining participants were dosed in groups of two using a sequential dosing strategy, and in part 3, the remaining six participants were dosed as a single second cohort.

Adults aged 18–65 years and weighing between 50 and <100 kg with no clinically significant abnormalities were eligible for inclusion. Individuals positive for HIV, SARS-CoV-2, and/or hepatitis C virus antibodies were excluded, along with those with evidence of hepatitis B virus infection (only those with both positive hepatitis B core antibody and positive hepatitis B surface antibody were included as these individuals were immune to hepatitis B virus). Individuals with uncontrolled hypertension, skin disorders or tattoos near the injection sites, or previous exposure to a monoclonal antibody or bNAb were not eligible for participation.

The study was performed in accordance with the International Council for Harmonization Good Clinical Practice guidelines and the ethical principles described in the Declaration of Helsinki. Protocols and informed consent forms were reviewed and pre-approved by Salus Institutional Review Board (Austin, TX). Written informed consent was obtained from all participants before initiation of any study procedures.

### Outcomes

Primary endpoints included the number and proportion of participants experiencing adverse events (AEs; grade ≥2) and serious AEs, and incidence of elevated liver parameters (grade ≥2) through Week 24. For parts 1 and 3 (SC dosing), the number and proportion of participants experiencing injection site reactions (ISRs) within 7 days of SC administration were also primary endpoints. AEs, including ISRs, were graded using the Division of AIDS criteria (version 2.1) (https://rsc.niaid.nih.gov/sites/default/files/daidsgradingcorrectedv21.pdf). Secondary endpoints included safety parameters (including ISR incidence, grade, and duration), PK parameters, and participant-reported outcomes. In parts 1 and 3 (SC dosing), ISR acceptability and proportion of participants bothered by injection site pain or local reactions were assessed on Days 2 and 7 post-dose using the Perception of Injection (PIN) Questionnaire with five-option scales: extremely/totally, very, moderately, a little, or not at all. The PIN Questionnaire was modified with consent from the Vaccinees’ Perception of Injection (VAPI), a validated instrument developed by Sanofi Pasteur (17). Post-infusion/injection pain perception was assessed in all participants using a numeric rating scale (NRS) on Days 1, 2, and 7 (from 0 = no pain to 10 = extreme pain). The detection of N6LS ADAs was an exploratory endpoint. Clinical laboratory assessments (i.e., hematology, clinical chemistry), electrocardiograms, and vital signs were monitored throughout the study.

### Pharmacokinetics

Blood samples were collected pre-dose; at infusion/injection start; at 4, 24, 48, and 72 hours post-infusion/injection; and at Weeks 1, 2, 3, 4, 8, 12, 16, 20, and 24. In parts 1 and 3, an additional sample was collected at the end of infusion. N6LS concentrations were determined using a validated method based on Protein A immunocapture and enzymatic digestion followed by liquid chromatography-tandem mass spectrometry analysis. The lower limit of quantification (LLOQ) was 1.00 µg/mL over a range of 1.00–100 µg/mL.

### Anti-drug antibodies

Serum samples collected at baseline (pre-dose) and at Weeks 4, 8, 12, 16, 20, and 24 were assessed for the presence of ADAs. A validated assay was used (an acid dissociation homogeneous bridging method on the Meso Scale Discovery platform, utilizing biotinylated N6LS as the capture reagent and ruthenylated N6LS as the detection reagent). A tiered assay approach was applied. First, samples were assessed in a screening assay, then positive samples were assessed in a confirmatory assay. Titer was also determined in samples that were positive in the confirmatory assay.

### Analyses

No formal hypotheses were statistically tested. The *n* = 8 sample size per dosing group was based on clinical and practical considerations and judged to be sufficient to assess safety and PK. The study population consisted of all participants who provided informed consent and were enrolled in the study; the safety and ADA populations consisted of those who received at least one dose of study drug; and the PK population included participants who had at least one post-dose PK concentration available. PK parameters were calculated based on actual sampling times using non-compartmental analysis with Phoenix WinNonlin (version 8.3, Certara, Princeton, NJ). Statistical outputs, including summary statistics, were derived using SAS (version 9.4; SAS Institute Inc, Cary, NC).

## RESULTS

### Study population

A total of 24 participants were enrolled across the three study parts and included in every analysis population. Mean age ranged from 39.9 to 42.8 years; 15 (63%) were female; 12 (50%) identified as White, 7 (29%) as Black or African American, and 8 (33%) as Hispanic or Latin American (Table 1). Overall, 1/24 (4%) participant was withdrawn before study completion on Day 84 (part 3 [SC dosing]; lost to follow-up), and 3/24 (13%) participants, all from part 3, had important protocol deviations (out-of-window visits, two of which were outside the ±7 day window). No protocol deviations were considered to have an impact on analyses.

**TABLE 1:** Participant demographics and baseline characteristics (study population)^*a*^

Parameter	Part 1	Part 2	Part 3
	N6LS 20 mg/kg SC + rHuPH20 2,000 U/mL (*N* = 8)	N6LS 60 mg/kg IV (*N* = 8)	N6LS 3,000 mg (~40 mg/kg) SC + rHuPH20 2,000 U/mL (*N* = 8)
Age, mean (SD), yr^*b*^	42.8 (7.8)	40.0 (14.5)	39.9 (10.6)
Male sex, *n* (%)	3 (38)	1 (13)	5 (63)
Race, *n* (%)			
Asian	2 (25)	1 (13)	1 (13)
Black or African American	1 (13)	2 (25)	4 (50)
Multiple races	0	1 (13)	0
White	5 (63)	4 (50)	3 (38)
Hispanic or Latin American ethnicity, *n* (%)	2 (25)	4 (50)	2 (25)
BMI, mean (SD), kg/m^2^	25.2 (3.5)	28.0 (3.5)	29.2 (4.8)

^*a*^ BMI, body mass index; IV, intravenous; N6LS, VH3810109; rHuPH20, recombinant human hyaluronidase PH20; SC, subcutaneous.

^*b*^ Age was imputed if the full date of birth was not provided.

### Safety outcomes

Safety outcomes are summarized in Table 2. No AEs led to withdrawal, and no serious AEs or deaths occurred. All 16 participants in the SC dosing groups reported ≥1 AE, and no relevant differences in overall AE incidence were observed between SC dosing groups. Only 1/8 (13%) participant in the IV dosing group reported AEs; none were ISRs, and all were grade 1 in intensity (*n* = 1 event of medical device site dermatitis at electrocardiogram electrode sites due to adhesive, *n* = 2 events of muscle tightness). The higher incidence of AEs with SC administration compared with IV was primarily driven by ISRs. One grade ≥ 2 non-ISR AE was reported across all dosing groups (grade 2 headache; *n* = 1, part 1 [SC dosing]). One participant reported non-ISR drug-related AEs across all dosing groups (grade 1 dyspepsia and flatulence; *n* = 1 each, part 3 [SC dosing]). No clinically significant safety trends in vital signs, electrocardiograms, or laboratory values were observed in any dosing group.

**TABLE 2:** Summary of AEs and ISRs across all doses (safety population)^*a*^^*,^b^*^

Participants, *n* (%)	Part 1	Part 2	Part 3	Total (*N* = 24)
	N6LS 20 mg/kg SC + rHuPH20 2,000 U/mL (*N* = 8)	N6LS 60 mg/kg IV (*N* = 8)	N6LS 3,000 mg (~40 mg/kg) SC + rHuPH20 2,000 U/mL (*N* = 8)	
Any AE	8 (100)	1 (13)	8 (100)	17 (71)
Grade 3 AEs	6 (75)	0	8 (100)	14 (58)
Grade ≥ 4 AEs	0	0	0	0
Serious AEs	0	0	0	0
Drug-related AEs	7 (88)	0	8 (100)	15 (63)
Grade ≥ 3 drug-related AEs (all ISRs)	6 (75)	0	8 (100)	14 (58)
AEs leading to withdrawal	0	0	0	0
Deaths	0	0	0	0
AEs occurring in >1 participant				
Injection site bruising	0	0	2 (25)	2 (8)
Injection site erythema	7 (88)	0	8 (100)	15 (63)
Injection site pain	1 (13)	0	2 (25)	3 (13)
Muscle tightness	1 (13)	1 (13)	0	2 (8)
Nasopharyngitis	0	0	2 (25)	2 (8)
Upper respiratory tract infection	2 (25)	0	1 (13)	3 (13)
Viral infection	1 (13)	0	1 (13)	2 (8)
ISRs (within 7 days of administration)	7 (88)	0	8 (100)	15 (63)
Grade 1	2 (25)	0	3 (38)	5 (21)
Injection site bruising	0	0	2 (25)	2 (8)
Injection site induration	0	0	1 (13)	1 (4)
Injection site pain	1 (13)	0	2 (25)	3 (13)
Injection site pruritus	1 (13)	0	0	1 (4)
Injection site warmth	0	0	1 (13)	1 (4)
Grade 2	2 (25)	0	2 (25)	4 (17)
Injection site erythema	2 (25)	0	2 (25)	4 (17)
Injection site pruritus	1 (13)	0	0	1 (4)
Grade 3	6 (75)	0	8 (100)	14 (58)
Injection site erythema^*c*^	6 (75)	0	8 (100)	14 (58)
ISR events, *n*	14	0	18	32

^*a*^ AE, adverse event; ISR, injection site reaction; IV, intravenous; N6LS, VH3810109; rHuPH20, recombinant human hyaluronidase PH20; SC, subcutaneous.

^*b*^ Note: Participants reporting ≥1 event were counted once at each level of summarization; therefore, the sum of AEs may be greater than reported at each level of summarization.

^*c*^ All grade 3 ISRs were injection site erythema based on size with no reports of complications (e.g., secondary infection, ulceration, phlebitis, sterile abscess, drainage, or symptoms causing an inability to perform usual social and functional activities).

All reported ISRs occurred in the SC dosing groups: 32 ISR events were reported by 15/16 (94%) participants (Table 2). All grade 3 ISRs (*n* = 17 events reported by *n* = 14 participants) were injection site erythema, with a mean duration of 2.9 days (part 1) and 5.7 days (part 3). In part 3 (N6LS 3,000 mg [~40 mg/kg] + rHuPH20), 3/8 (38%) participants experienced grade 3 injection site erythema with larger peak surface area (≥240 cm^2^) compared with other participants in the group and with those in part 1 (40–165 cm^2^); in these participants, erythema reached peak area measurements on Day 3 or 4, followed by large surface area reductions the day after peak area was reached. No apparent trends in demographics were observed among the three participants who experienced grade 3 injection site erythema. All ISRs resolved without sequelae, treatment, or complications. The majority of ISRs resolved within 7 days; only one ISR took longer to resolve (injection site erythema; part 3, 27 days). Biphasic injection site erythema, where an initial erythema event first diminished in size and then later increased in size, was reported in 2/8 (25%) participants in part 1 and 4/8 (50%) participants in part 3. The onset of the second phase occurred within 20–47 hours after injection, with a total duration of erythema ranging from 3 to 8 days.

### Participant-reported outcomes

#### Numeric rating scale

In the IV dosing group, mean NRS scores for post-infusion/injection pain were 0.50–0.88 on Days 1, 2, and 7 (Table 3). Maximum reported score was <5 (5 = moderate pain) at all time points (3 on Day 1; 4 on Days 2 and 7). In the SC dosing groups, mean NRS scores were 3.63 at all time points in part 1 and 2.75, 0.63, and 0.38 on Days 1, 2, and 7, respectively, in part 3. Maximum scores reported in part 1 were >5 (5 = moderate pain) at all time points (7 on Days 1 and 2; 8 on Day 7); in part 3, the maximum score was 6 on Day 1 and 2 on Days 2 and 7. Minimum reported score was 0 (no pain) at each time point across both SC and IV dosing groups.

**TABLE 3:** Numeric rating scale assessment (safety population)^*a*^

NRS score	Part 1	Part 2	Part 3
	N6LS 20 mg/kg SC + rHuPH20 2,000 U/mL (*N* = 8)	N6LS 60 mg/kg IV (*N* = 8)	N6LS 3,000 mg (~40 mg/kg) SC + rHuPH20 2,000 U/mL (*N* = 8)
Day 1			
Mean (SD)	3.63 (2.33)	0.50 (1.07)	2.75 (2.55)
Median	3.5	0.0	2.0
Minimum	0 [*n* = 1]	0 [*n* = 6]	0 [*n* = 2]
Maximum	7 [*n* = 1]	3 [*n* = 1]	6 [*n* = 2]
Day 2			
Mean (SD)	3.63 (2.62)	0.88 (1.64)	0.63 (0.74)
Median	3.5	0.0	0.5
Minimum	0 [*n* = 1]	0 [*n* = 6]	0 [*n* = 4]
Maximum	7 [*n* = 2]	4 [*n* = 1]	2 [*n* = 1]
Day 7			
Mean (SD)	3.63 (2.93)	0.50 (1.41)	0.38 (0.74)
Median	3.5	0.0	0.0
Minimum	0 [*n* = 1]	0 [*n* = 7]	0 [*n* = 6]
Maximum	8 [*n* = 1]	4 [*n* = 1]	2 [*n* = 1]

^*a*^ IV, intravenous; N6LS, VH3810109; NRS, numeric rating scale; rHuPH20, recombinant human hyaluronidase PH20; SC, subcutaneous.

#### Perception of Injection Questionnaire (subcutaneous administration)

**Fig 1:**
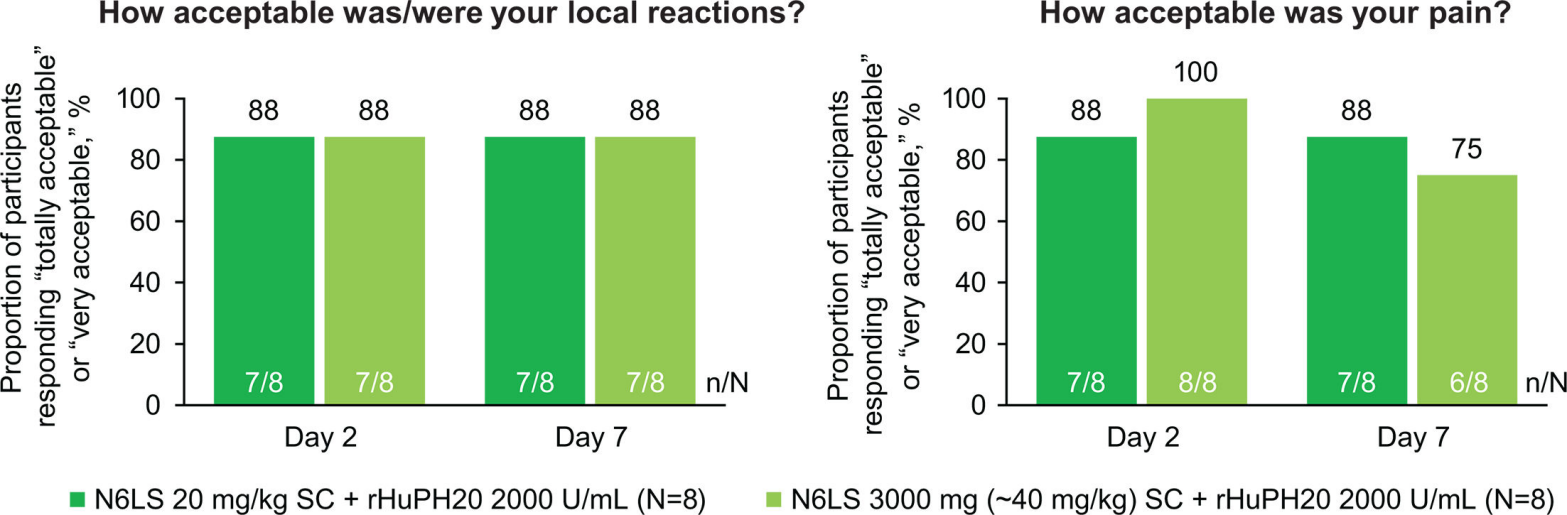
Acceptability of SC injections among participants receiving N6LS SC + rHuPH20 using the Perception of Injection Questionnaire. N6LS, VH3810109; rHuPH20, recombinant human hyaluronidase PH20; SC, subcutaneous.

At Days 2 and 7 post-dose, all participants in the SC dosing groups reported that local reactions and pain from injections were acceptable, with the majority (75–100%) reporting “totally” or “very” acceptable (Fig. 1) and all others reporting “moderately” (0–25%) or “a little” (0–13%) acceptable at each time point. In part 1 (20 mg/kg), no participants reported being affected by local reactions or pain when falling asleep, changing position at night, moving, or walking on Days 2 and 7 post-dose. In part 3 (3,000 mg [~40 mg/kg]), most (75% [6/8] to 100% [8/8]) participants reported feeling “not at all” affected by local reactions or pain. On Day 2 post-dose, one participant reported feeling “a little” affected by local reactions when falling asleep, and three participants felt affected by local reactions (“a little,” *n* = 1; “moderately,” *n* = 1) or pain (“a little,” *n* = 1) when changing positions at night. On Day 7 post-dose, two participants were affected by local reactions when falling asleep, two were affected by pain when changing positions at night, and one was affected by local reactions and pain when moving or walking (each rated as “a little” affected).

### Pharmacokinetics

**Fig 2:**
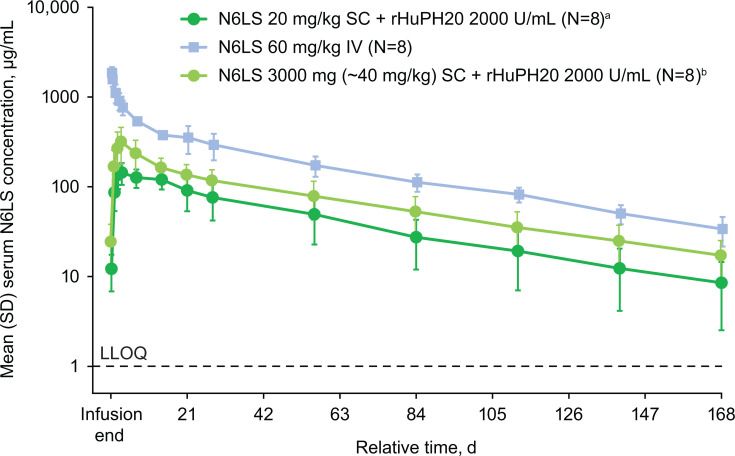
Serum concentrations over time in participants receiving a single dose of N6LS. ^a^Included values imputed as 0 for being below LLOQ (1.00 µg/mL; *n* = 1 value each for Days 56, 84, 112, and 140 and *n* = 2 values for Day 168). ^b^Data were available for 7/8 participants on Days 21, 56, 112, 140, and 168. IV, intravenous; LLOQ, lower limit of quantification; N6LS, VH3810109; rHuPH20, recombinant human hyaluronidase PH20; SC, subcutaneous.

Serum N6LS PK parameters for all participants are presented in Table 4, and N6LS concentration-time profiles are presented in Fig. 2. A single dose of 20 mg/kg and 3,000 mg (~40 mg/kg) of N6LS administered SC with rHuPH20 gave arithmetic mean (95% CI) maximum concentrations of 148 (117–179) and 321 (202–441) µg/mL, respectively, and arithmetic mean (95% CI) area under the serum concentration-time curve from time 0 extrapolated to infinity values of 7,587 (4,789–10,380) and 12,840 (8,883–16,800) day·µg/mL, respectively. After a single 60 mg/kg IV dose of N6LS, arithmetic mean (95% CI) maximum concentration was 1,864 (1,620–2,107) µg/mL, and arithmetic mean (95% CI) area under the serum concentration-time curve from time 0 extrapolated to infinity was 31,560 (27,620–35,490) day·µg/mL. Across all parts, median terminal half-life ranged from 43 to 47 days.

**TABLE 4:** N6LS serum PK parameters by study part (PK population)^*a*^

Arithmetic mean (95% CI)^*b*^	Part 1	Part 2	Part 3
	N6LS 20 mg/kg SC + rHuPH20 2,000 U/mL (*N* = 8)	N6LS 60 mg/kg IV (*N* = 8)	N6LS 3,000 mg (~40 mg/kg) SC + rHuPH20 2,000 U/mL (*N* = 8)
AUC_0-∞_, day·µg/mL	7,587 (4,789–10,380)	31,560 (27,620–35,490)	12,840 (8,883–16,800)
Cmax, µg/mL	148 (117–179)	1,864 (1,620–2,107)	321 (202–441)
tmax, day^*c*^	3.00 (2.00–13.00)	0.05 (0.05–0.05)	3.01 (2.01–3.01)
t_1/2_, day^*d*^	46.1 (23.4–52.9)	47.3 (40.9–53.1)	43.3 (34.1–55.8)
CL or CL/F, L/day^*e*^^,^^*f*^	0.277 (0.121–0.917)^*g*^	0.136 (0.110–0.157)	0.266 (0.134–0.513)

^*a*^ ADA, anti-drug antibody; AUC_0-∞_, area under the serum concentration-time curve from time 0 extrapolated to infinity; CI, confidence interval; CL or CL/F, apparent total body clearance; Cmax, maximum observed serum concentration; IV, intravenous; max, maximum; min, minimum; N6LS, VH3810109; PK, pharmacokinetic; rHuPH20, recombinant human hyaluronidase PH20; SC, subcutaneous; t_1/2_, terminal half-life; tmax, time of Cmax.

^*b*^ Unless otherwise specified.

^*c*^ Median (min-max).

^*d*^ Median (95% CI).

^*e*^ CL for IV administration, CL/F for SC administration.

^*f*^ Arithmetic mean (min-max).

^*g*^ CL/F was 0.917 L/day in the participant with treatment-emergent ADA beginning at Week 4 (titer range, 40–5,120) and 0.359 L/day in the participant with treatment-emergent ADA beginning at Week 12 (titer range, 80–640).

### Anti-drug antibodies

None of the 24 participants had ADAs in the pre-dose sample. All eight participants in the N6LS 60 mg/kg IV dosing group were ADA-negative through Week 24 post-dose. In the N6LS 20 mg/kg SC + rHuPH20 dosing group, 3/8 (38%) participants had treatment-emergent ADAs, and in the N6LS 3,000 mg (~40 mg/kg) SC + rHuPH20 dosing group, 2/8 (25%) had treatment-emergent ADAs, giving an overall treatment-emergent ADA incidence of 5/16 (31%). In three of these five participants (*n* = 1 in part 1 [20 mg/kg]; *n* = 2 in part 3 [3,000 mg]), samples were only ADA-positive at later time points (Weeks 20 and 24), titers were low (<40), and there was no impact of ADAs on PK. In the remaining two participants in part 1 (20 mg/kg), who had relatively high titers (up to 5,120 and 640, respectively), time to onset of ADAs was Week 4 (i.e., the first post-dose ADA sample) and Week 12, respectively, with titers peaking at Week 20 and then decreasing again through Week 24 in both participants. There was an observed decrease in PK concentrations and an increase in N6LS clearance (CL/F) in the one participant who had ADAs from Week 4 (Table 4). The CL/F for the other participant was within the range of CL/F values observed across ADA-negative participants; therefore, no clear impact of ADAs on PK in this participant was evident.

There was no apparent association between the presence of ADAs and the occurrence of ISRs; not all participants who were ADA-positive had ISRs, and those participants with ADAs who had ISRs did not experience ISRs of the highest grade or the longest duration.

## DISCUSSION

The SPAN study assessed the safety, tolerability, PK, and incidence of ADAs for the bNAb N6LS administered as a single SC or IV dose to generate data to support studies of multiple-dose N6LS in adults with HIV-1. A single dose of N6LS administered SC (20 or ~40 mg/kg) + rHuPH20 or IV (60 mg/kg) in adults without HIV had a favorable safety profile and was well tolerated.

No serious AEs, deaths, AEs leading to withdrawal, or clinically significant changes from baseline in other safety parameters were reported across all doses in this study. This finding is consistent with phase 1 studies of other bNAbs targeting the CD4-binding site, including VRC01 dosed at 5–40 mg/kg IV and 5 mg/kg SC in participants without HIV and 3BNC117 dosed at 1–30 mg/kg IV in people with or without HIV-1, which also reported mild or moderate AEs and no serious AEs (3, 18).

Larger drug volumes can generally be introduced IV compared with SC, as the latter environment consists of polymerized extracellular matrix proteins that resist fluid flow (15, 19). The use of rHuPH20 to increase SC tissue permeability is approved to facilitate the dispersion and absorption of other drugs (14), and co-administration with other monoclonal antibodies has been approved for volumes of up to 600 mL per injection site (20). The injection volumes used in SPAN were ~15 mL for N6LS 20 mg/kg and ~30 mL for N6LS 3,000 mg (~40 mg/kg) when co-administered with rHuPH20.

Overall AE incidence was higher with SC administration of N6LS compared with IV administration, primarily due to the high incidence of ISRs with SC dosing. Other studies assessing co-administration of rHuPH20 with different therapeutics also reported higher proportions of people experiencing administration site reactions with SC vs IV dosing (range, 7–91% vs 1–35%) and concluded that AEs were usually due to either the safety profile of the therapeutic or the rapid administration of a large SC bolus, permitted through the use of rHuPH20 (15). All grade 3 AEs reported in SPAN were injection site erythema, and 38% of participants across parts 1 and 3 experienced biphasic erythema. All ISR AEs resolved without sequelae or treatment, all but one of them within 7 days, and participants receiving N6LS SC dosing reported that pain and local reactions from the injection were acceptable. With IV administration, the only AEs reported were a skin reaction due to electrocardiogram monitoring electrodes and two AEs of muscle tightness; all were grade 1 in severity. Though more frequent and/or higher-grade AEs were observed with SC dosing, N6LS was well tolerated across all doses and routes of administration.

N6LS PK profiles after IV and SC administration were as expected for a bNAb, with a long terminal half-life (43–47 days), and were consistent with outcomes from the phase 1 study of N6LS administered either IV or SC in participants without HIV (7, 21). Due to the well-characterized PK profile of rHuPH20 when co-administered with other injectable drugs, we did not assess rHuPH20 PK parameters.

Immunogenicity, characterized by the formation of ADAs, commonly occurs after administration of biologics (22). The presence of ADAs may impact PK by accelerating drug clearance (22). Of the 24 participants in SPAN, none had pre-existing ADAs (all were ADA-negative in the pre-dose sample). All eight participants in the N6LS 60 mg/kg IV dosing group were ADA-negative through Week 24 post-dose. Incidence of treatment-emergent ADAs was 31% (5/16) after SC administration, and drug clearance was accelerated in only one of these five participants. Previous ADA assessments of N6LS in people without HIV demonstrated no evidence of ADA development over 12 or 24 weeks (16, 21). Factors that can impact the incidence of ADAs include route of administration (with IV expected to have the lowest incidence), participant population, and dose and frequency of administration (22). Further information on the immunogenicity profile of N6LS will be obtained from the phase 2a BANNER study, in which single doses of N6LS were administered IV and SC to adults with HIV-1 with no prior exposure to ART, and in the ongoing phase 2b EMBRACE study (ClinicalTrials.gov, NCT05996471), in which IV or SC doses are being administered every 4 months to adults with HIV-1 who are virologically suppressed.

Overall, N6LS administered IV or SC + rHuPH20 demonstrated a favorable safety profile and was well tolerated, with participants rating both pain and local reactions from SC injections as acceptable. PK were as expected for a bNAb, and the incidence of ADAs was low (0/8 [0%] after IV dosing and 5/16 [31%] after SC dosing), with no impact on PK in all but one participant. Taken together, data from this study support the exploration of these doses in adults with HIV-1. Given that these are relatively high doses, there is also potential for N6LS to support less frequent dosing in adults with HIV-1 (dosing once every ≥4 months), to provide an ultra-long-acting HIV-1 therapy option. Results from SPAN support the ongoing clinical development of N6LS in adults with HIV-1, namely, the phase 2a BANNER study in adults with no prior exposure to ART, followed by subsequent assessment of N6LS 60 mg/kg IV and 3,000 mg (~40 mg/kg) SC + rHuPH20 doses in the ongoing phase 2b EMBRACE study in adults with virologic suppression.

## Data Availability

Anonymized individual participant data and study documents can be requested for further research from www.clinicalstudydatarequest.com.

## References

[1] Mahomed S, Garrett N, Baxter C, Abdool Karim Q, Abdool Karim SS. 2021. Clinical trials of broadly neutralizing monoclonal antibodies for human immunodeficiency virus prevention: a review. J Infect Dis 223:370–380. doi:10.1093/infdis/jiaa377 32604408 PMC8508778

[2] Gaebler C, Nogueira L, Stoffel E, Oliveira TY, Breton G, Millard KG, Turroja M, Butler A, Ramos V, Seaman MS, Reeves JD, Petroupoulos CJ, Shimeliovich I, Gazumyan A, Jiang CS, Jilg N, Scheid JF, Gandhi R, Walker BD, Sneller MC, Fauci A, Chun T-W, Caskey M, Nussenzweig MC. 2022. Prolonged viral suppression with anti-HIV-1 antibody therapy. Nature 606:368–374. doi:10.1038/s41586-022-04597-1 35418681 PMC9177424

[3] Caskey M, Klein F, Lorenzi JCC, Seaman MS, West AP Jr, Buckley N, Kremer G, Nogueira L, Braunschweig M, Scheid JF, Horwitz JA, Shimeliovich I, Ben-Avraham S, Witmer-Pack M, Platten M, Lehmann C, Burke LA, Hawthorne T, Gorelick RJ, Walker BD, Keler T, Gulick RM, Fätkenheuer G, Schlesinger SJ, Nussenzweig MC. 2015. Viraemia suppressed in HIV-1-infected humans by broadly neutralizing antibody 3BNC117. Nature 522:487–491. doi:10.1038/nature14411 25855300 PMC4890714

[4] Caskey M, Schoofs T, Gruell H, Settler A, Karagounis T, Kreider EF, Murrell B, Pfeifer N, Nogueira L, Oliveira TY, et al.. 2017. Antibody 10-1074 suppresses viremia in HIV-1-infected individuals. Nat Med 23:185–191. doi:10.1038/nm.4268 28092665 PMC5467219

[5] Corey L, Gilbert PB, Juraska M, Montefiori DC, Morris L, Karuna ST, Edupuganti S, Mgodi NM, deCamp AC, Rudnicki E, et al.. 2021. Two randomized trials of neutralizing antibodies to prevent HIV-1 acquisition. N Engl J Med 384:1003–1014. doi:10.1056/NEJMoa2031738 33730454 PMC8189692

[6] Crowell TA, Colby DJ, Pinyakorn S, Sacdalan C, Pagliuzza A, Intasan J, Benjapornpong K, Tangnaree K, Chomchey N, Kroon E, et al.. 2019. Safety and efficacy of VRC01 broadly neutralising antibodies in adults with acutely treated HIV (RV397): a phase 2, randomised, double-blind, placebo-controlled trial. Lancet HIV 6:e297–e306. doi:10.1016/S2352-3018(19)30053-0 31000477 PMC6693657

[7] Caskey M. 2020. Broadly neutralizing antibodies for the treatment and prevention of HIV infection. Curr Opin HIV AIDS 15:49–55. doi:10.1097/COH.0000000000000600 31764199 PMC7340121

[8] Huang J, Kang BH, Ishida E, Zhou T, Griesman T, Sheng Z, Wu F, Doria-Rose NA, Zhang B, McKee K, et al.. 2016. Identification of a CD4-binding-site antibody to HIV that evolved near-pan neutralization breadth. Immunity 45:1108–1121. doi:10.1016/j.immuni.2016.10.027 27851912 PMC5770152

[9] Julg B, Pegu A, Abbink P, Liu J, Brinkman A, Molloy K, Mojta S, Chandrashekar A, Callow K, Wang K, Chen X, Schmidt SD, Huang J, Koup RA, Seaman MS, Keele BF, Mascola JR, Connors M, Barouch DH. 2017. Virological control by the CD4-binding site antibody N6 in simian-human immunodeficiency virus-infected rhesus monkeys. J Virol 91:e00498-17. doi:10.1128/JVI.00498-17 28539448 PMC5533891

[10] Edwards AY, Ashraf W, Zweers T, Brown K, Losos J, Leone P, Gartland M, Wannamaker P, Lataillade M, Gandhi Y. 2023. Pharmacokinetics/Pharmacodynamics and virological activity of VH3810109 (N6LS) in antiretroviral-naive viremic adults from the phase 2a BANNER study. Abstr 19th European AIDS Conference; abstr eP.A.099

[11] Leone P, Cahn P, Rolle C-P, Klein M, Bettacchi C, Losos J, Griesel R, Warwick-Sanders M, Win B, Gandhi Y, D’Agostino R, Wannamaker P, Abberbock J, Lataillade M. 2023. Safety and tolerability of VH3810109 (N6LS) among antiretroviral therapy–naive adults living with HIV-1: results from the monotherapy phase of the phase 2a BANNER study. Abstr 19th European AIDS Conference; abstr PS8.O5

[12] Leone P, Ferro A, Rolle C-P, Lupo S, McGowan J, Klein M, Cahn P, Benson P, Sanchez M, Bettacchi C, Schneider S, Wannamaker P, Win B, Abberbock J, Baker M, Wilches V, Bentley D, Gartland M, Lataillade M, Losos J. 2022. VH3810109 (N6LS) reduces viremia across a range of doses in ART-naive adults living with HIV: proof of concept achieved in the phase IIa BANNER (207959, NCT04871113) study. Abstr HIV Drug Therapy Glasgow; abstr O34

[13] Usach I, Martinez R, Festini T, Peris J-E. 2019. Subcutaneous injection of drugs: literature review of factors influencing pain sensation at the injection site. Adv Ther 36:2986–2996. doi:10.1007/s12325-019-01101-6 31587143 PMC6822791

[14] Halozyme, Inc. 2024. Hylenex recombinant [prescribing information]. Halozyme, Inc, San Diego, CA.

[15] Knowles SP, Printz MA, Kang DW, LaBarre MJ, Tannenbaum RP. 2021. Safety of recombinant human hyaluronidase PH20 for subcutaneous drug delivery. Expert Opin Drug Deliv 18:1673–1685. doi:10.1080/17425247.2021.1981286 34585991

[16] Wu RL, Houser KV, Happe M, Gaudinski MR, Awan SF, Widge AT, Holman LA, Saunders J, Buettner C, Lin BC, Castro M, Capparelli EV, Seder RA, Koup RA, Dropulic LK. 2023. N6LS with rHuPH20 enables safe high dose monoclonal antibody subcutaneous delivery. Abstr 30th Conference on Retroviruses and Opportunistic Infections; abstr 499

[17] Chevat C, Viala-Danten M, Dias-Barbosa C, Nguyen VH. 2009. Development and psychometric validation of a self-administered questionnaire assessing the acceptance of influenza vaccination: the Vaccinees’ Perception of Injection (VAPI) questionnaire. Health Qual Life Outcomes 7:21. doi:10.1186/1477-7525-7-21 19261173 PMC2660294

[18] Ledgerwood JE, Coates EE, Yamshchikov G, Saunders JG, Holman L, Enama ME, DeZure A, Lynch RM, Gordon I, Plummer S, et al.. 2015. Safety, pharmacokinetics and neutralization of the broadly neutralizing HIV-1 human monoclonal antibody VRC01 in healthy adults. Clin Exp Immunol 182:289–301. doi:10.1111/cei.12692 26332605 PMC4636891

[19] Davis JD, Bravo Padros M, Conrado DJ, Ganguly S, Guan X, Hassan HE, Hazra A, Irvin SC, Jayachandran P, Kosloski MP, Lin K-J, Mukherjee K, Paccaly A, Papachristos A, Partridge MA, Prabhu S, Visich J, Welf ES, Xu X, Zhao A, Zhu M. 2024. Subcutaneous administration of monoclonal antibodies: pharmacology, delivery, immunogenicity, and learnings from applications to clinical development. Clin Pharmacol Ther 115:422–439. doi:10.1002/cpt.3150 38093583

[20] Takeda Pharmaceuticals USA, Inc. 2024. HyQvia [prescribing information]. Takeda Pharmaceuticals USA, Inc, Lexington, MA.

[21] Widge AT, Houser KV, Gaudinski MR, Carter C, Hickman SP, Saunders J, Holman L, Gordon I, O’Connell S, Capparelli EV, Connors M, Chen GL, Koup RA, Mascola JR. 2020. Phase I dose-escalation study of human monoclonal antibody N6LS in healthy adults. Abstr 27th Conference on Retroviruses and Opportunistic Infections; abstr 508

[22] US Department of Health and Human Services, Food and Drug Administration, Center for Drug Evaluation and Research, Center for Biologics Evaluation and Research. 2014. Immunogenicity assessment for therapeutic protein products: guidance for industry. Food and Drug Administration, Silver Spring, MD.

